# Intra-arterial anaesthetics for pain control in arterial embolisation procedures: a systematic review and meta-analysis

**DOI:** 10.1186/s42155-020-00198-z

**Published:** 2021-01-05

**Authors:** Taha Hanif Shiwani, Hunain Shiwani

**Affiliations:** 1 Imperial College London, London, UK; 2 Leeds Teaching Hospitals NHS Trust, Leeds, West Yorkshire UK

**Keywords:** Systematic review, Meta-analysis, Embolisation, Embolization, Arterial, Intraarterial, Anaesthetic, Anaesthesia, Pain control, Pain management, Interventional radiology

## Abstract

**Purpose:**

A systematic review to determine the effectiveness of intra-arterial anaesthetics on post- operative pain and opioid analgesia requirements in arterial embolisation procedures.

**Materials and methods:**

A systematic review of the literature was performed (Embase, PubMed, MEDLINE and the Cochrane Library) from inception to 10th August 2020. Randomised controlled trials (RCTs) and cohort studies that utilised intra-arterial anaesthesia during an embolisation procedure for the purposes of pain control were included. Eligibility was assessed by two investigators independently.

**Results:**

Eight hundred fifty-nine candidate articles were identified, and 9 studies met the inclusion criteria (6 RCTs and 3 retrospective cohort studies). Four studies were of hepatic chemoembolisation and 5 were of uterine artery embolisation.

Five hundred twenty-nine patients were treated in total. All studies used lidocaine as the anaesthetic with doses ranging from 20 to 200 mg, and the anaesthetic was delivered varyingly before, during or after embolisation. Pain intensity was converted to a numeric scale from 0 to 10, and opioid doses were converted to milligram morphine equivalent doses. A random-effects meta-analysis model was used to analyse the results of RCTs, and the results of cohort studies were summarised with a narrative synthesis. The meta-analyses suggested that pain scores were reduced by a mean of 1.02 (95% CI − 2.34 to 0.30; *p* = 0.13) and opioid doses were reduced by a mean of 7.35 mg (95% CI, − 14.77, 0.06; *p* = 0.05) in the intervention group however neither finding was statistically significant. No serious adverse events were reported.

**Conclusion:**

Intra-arterial anaesthetic may slightly reduce pain intensity and post-operative opioid consumption following embolisation, however the results are not statistically significant. There is very limited data available on the effect of anaesthetic on length of hospital admission. Whilst no serious adverse events were reported, there are some concerns regarding the effect of lidocaine on the technical success of embolisation procedures that preclude our recommendation for routine use in embolisation procedures.

High quality randomised controlled trials are required to elucidate the dose-response effect of lidocaine on opioid consumption and pain following embolisation, particularly in the first few hours post-operatively, as well as effects on duration of hospital stay.

**Supplementary Information:**

The online version contains supplementary material available at 10.1186/s42155-020-00198-z.

## Background

Arterial embolisation is currently best practice for a variety of disease processes with expanding and increasingly novel indications. However, embolisation procedures can often be complicated by significant post-operative pain due to the ischaemia and subsequent inflammation they induce in downstream tissues (Spencer et al. 2013).

Pain post embolisation is most clearly documented in the context of trans-arterial chemoembolisation (TACE) for HCC (Mason et al. 2015) and uterine fibroid embolisation (UFE) (Edwards et al. 2007). Post-operative pain has been strongly implicated in increasing a patient’s length of hospital stay and risk of overnight and recurrent admission following these procedures (Leung et al. 2001) (Spencer et al. 2013).

Protocols for pain control following arterial embolisation vary drastically between procedures and between institutions, however, most rely on traditional methods of patient controlled opioid analgesia (PCA) or intravenous (IV) opioid medication pre/post-operatively before transitioning patients on to oral opioids, non-steroidal anti-inflammatory drugs and/or paracetamol. Whilst effective, opioid medications are associated with significant adverse effects, most commonly nausea/vomiting and cognitive dysfunction (Swegle and Logemann 2006). Consequently, alternative measures for pain control have been proposed including local nerve blocks (e.g superior hypogastric nerve block for UFE) (Spencer et al. 2013) and intra-arterial administration of anaesthetic in the peri-procedural period.

The latter has been reported as a simple and transferrable intervention to reduce pain scores, reliance on opioid medication and recovery time following embolisation, however, there is no consensus in the literature regarding its safety and effectiveness. The potential mechanism of action is also unclear, with a suggestion that anaesthetics may diffuse into the arterial wall and exert a prolonged effect on local tissues following embolisation (Hartnell et al. 1999). This is thought to be due to reduced metabolism of the anaesthetic following occlusion of the local blood supply as well as decreased washout of the drug.

In this review, we aimed to systematically review and analyse the effectiveness of intra-arterial anaesthetics on reducing post-operative pain and opioid analgesia requirements in arterial embolisation procedures, with a secondary aim to determine the effect on length of hospital stay.

## Main text

### Methods

This study was registered on PROSPERO (CRD42020176020). A systematic review of the literature was performed (Embase, PubMed, MEDLINE and the Cochrane Library) from inception to 10th August 2020. A pre-determined search strategy was used with appropriate key words and Boolean operators (Additional file 1: Appendix 1).

Records obtained by the search strategy were aggregated into RefWorks. Duplicate records were identified and discarded. The abstracts and titles of all records were screened to exclude conference papers, reviews, case-series, articles that were not published in English, and articles that did not make reference to intra-arterial delivery of an anaesthetic for pain control in an embolisation procedure. The full texts of remaining articles were then reviewed against pre-defined eligibility criteria. Randomised trials and non-randomised prospective or retrospective cohort studies were included if they reported either a pain score or post-procedural analgesia requirements up to 48 h after an embolisation procedure. The following studies were excluded: case reports or case-series, those including children (< 16 yrs. of age), those not using an anaesthetic, those without a control arm (either placebo or no treatment), and those without a full-text article published in English.

The screening and selection processes were conducted independently by two investigators including a senior interventional radiology registrar.

#### Data extraction

The following data was obtained from the published report of each study and collated into a Microsoft Excel spreadsheet: study design; demographic details; indication for embolisation, method of embolisation; timing, type and dose of anaesthetic delivered; details of control arm; post-procedural pain score and/or analgesia requirements; length of hospital stay (where recorded); and complications/adverse events.

Post-procedural pain scores were standardised to a numerical rating scale (NRS) score from 0 to 10. Studies that did not include quantitative pain scores were excluded from the meta-analysis. A VAS score between 0 to 10 cm was considered to be equivalent to a numerical rating scale (NRS) score of 0 to 10 as they have been shown to be strongly correlated (Downie et al. 1978). Where pain intensity was measured using a visual analogue scale (VAS) of 0 to 100 cm, the VAS scores were converted to a score between 0 to 10 by dividing by 10.

Similarly, where required, post-procedural analgesia requirements were converted to milligram morphine equivalent doses using conversion ratios described in the literature. These included the following: 10:1 for IV meperidine: IV morphine (Pereira et al. 2001), 1:1 for IV nalbuphine: IV morphine (Zeng et al. 2015), 1:5 for IV hydromophone: IV morphine (Patanwala et al. 2007).

#### Risk of bias assessment

Each study included in the review was subjected to a risk of bias assessment. These were conducted independently by two investigators, with disagreements being resolved through discussion.

The risk of bias in randomised controlled trials (RCTs) was assessed using the Risk of Bias 2 (RoB 2) Tool developed by the Cochrane Collaboration (Sterne et al. 2019). This assesses the risk of biases in the following domains: 1) bias arising from the randomization process; 2) bias due to deviations from intended interventions; 3) bias due to missing outcome data; 4) bias in measurement of the outcome; 5) bias in selection of the reported result. Using the RoB 2 Tool, a judgement of “low risk”, “some concerns” or “high risk” was made for the risk of bias in each domain, allowing an overall risk of bias to be generated for each study using the tools algorithm.

The risk of bias in non-randomised cohort studies was assessed using the Newcastle-Ottawa Scale (NOS) (Wells et al. 2012). This considers the following study characteristics: 1) participant selection; 2) comparability of cohorts 3) outcome measurement. For ease of presentation, the NOS score was translated to a quality judgement of “good”, “fair” or “poor” as outlined by the Agency for Healthcare Research and Quality (AHRQ) using AHRQ conversion thresholds (Agency for Healthcare Research and Quality 2014).

#### Data analysis & synthesis

Results for all outcomes were expressed as means +/− standard deviations where possible. The formulas recommended by the Cochrane Handbook for Systematic Reviews of Interventions (Higgins et al. 2019) were used to calculate the standard deviation from confidence intervals or standard errors where necessary (Additional file 1: Appendix 2). If studies had more than one treatment arm, the groups were combined as recommended by the Cochrane Handbook (Higgins et al. 2019) (Additional file 1: Appendix 3).

The results of the RCTs were synthesised using a random-effects meta-analysis model to generate pooled mean differences for each primary outcome measurement, and a z-test was used to determine generate a *p*-value and determine statistical significance. A random-effects model was used as there was significant methodological and clinical heterogeneity in included studies. Mean difference was felt to be an appropriate summary statistic as all pain scores and opioid requirements were converted to the same scales. A forest plot was generated for each primary outcome measurement, including post-embolisation pain scores and opioid requirements. Where multiple pain scores were provided in a study, the mean pain score in the first 24 h post-procedure was selected for the forest plot, as it was the most consistently measured statistic and clinically influences the need for overnight hospital admission. Similarly, where multiple values for post-procedural opioid requirements were provided, the total dose of opioid administered post-procedure was included in the forest plot as it was the most consistently measured statistic. Statistical heterogeneity for all outcomes was quantified using the I^2^ statistic as described in the Cochrane Handbook (Higgins et al. 2019), and all analyses were conducted using RevMan 5 software. As only a small number of studies were ultimately included in the analyses, subgroup analyses and sensitivity analyses were not performed.

The results of the cohort studies were summarised separately using a narrative synthesis effect due to the variability in their outcome effect measures and use of statistical tests.

This systematic review was conducted using the guidelines documented in the 2009 PRISMA checklist (Moher et al. 2009).

## Results

### Search results

**Fig. 1:**
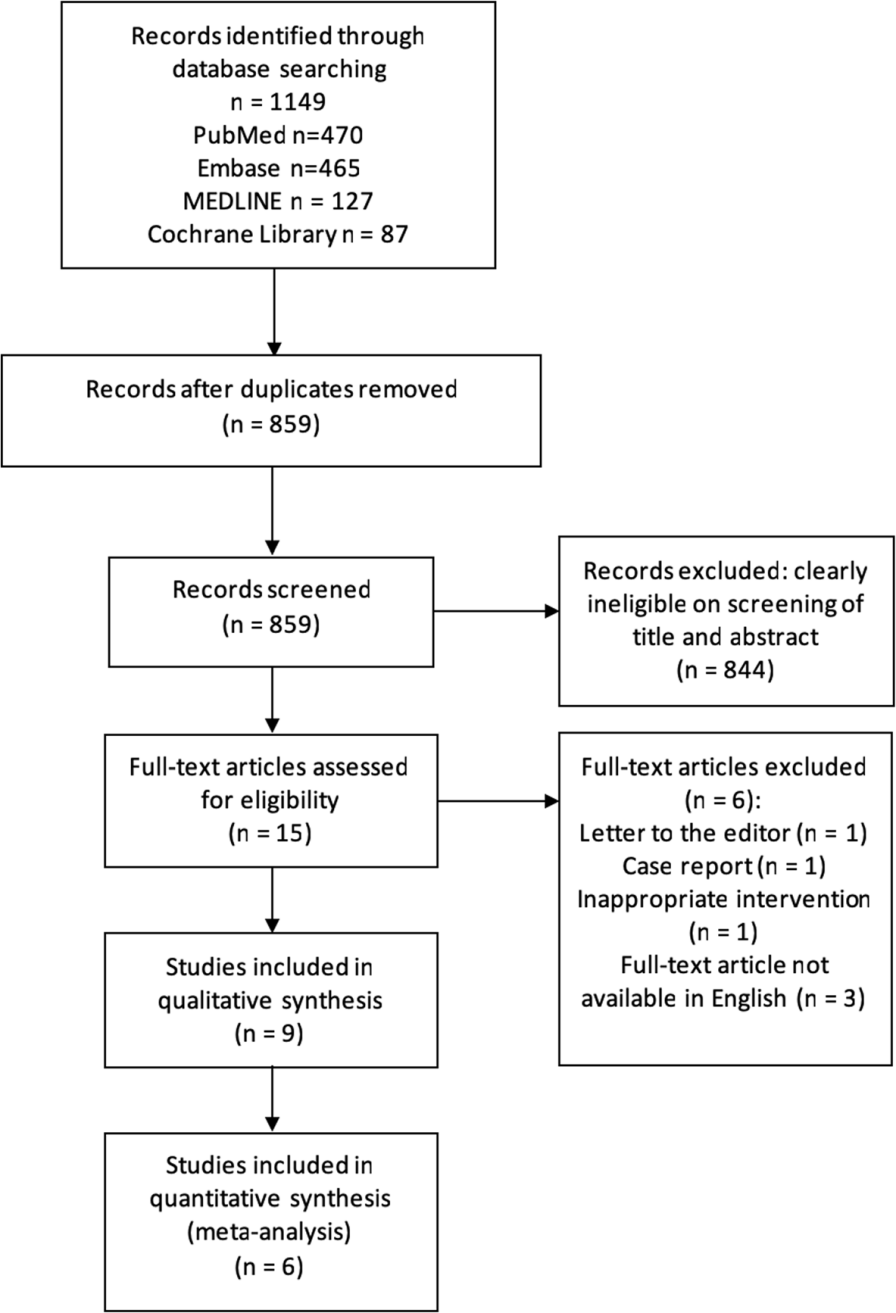
A PRISMA flow diagram outlining each phase of the systematic review

The search strategy yielded 1149 records. Of those, 290 were discarded as duplicates and 844 were discarded upon screening of their abstract and title. The full text of the remaining 15 records were then reviewed, revealing 9 eligible studies. This is summarised in detail using the PRISMA flowchart in Fig. 1.

### Characteristics of included studies

Nine studies reported a total of 548 embolisation procedures (316 intervention and 232 controls) in 529 participants.

Six studies were RCTs (Abusedera et al. 2014; Duvnjak and Andersen 2020; Keyoung et al. 2001; Lee et al. 2001; Noel-Lamy et al. 2017; Zhan et al. 2005) and three were cohort studies: two of mixed-design (control group studied retrospectively, intervention group studied prospectively), (Molgaard et al. 1990; Hartnell et al. 1999), and one retrospective cohort study (Katsumori et al. 2020).

Five studies investigated UFE (Duvnjak and Andersen 2020; Keyoung et al. 2001; Katsumori et al. 2020; Noel-Lamy et al. 2017; Zhan et al. 2005), two studies investigated hepatic TACE for the treatment of HCC alone (Abusedera et al. 2014; Lee et al. 2001) and two studies investigated hepatic TACE for HCC and metastatic disease (Molgaard et al. 1990, Hartnell et al. 1999). This is summarised in Table 1.

**Table 1:** A table summarising the studies included in this review and the embolization procedures that they investigated

	Randomised Controlled Trials (RCTs)	Cohort Studies
**Uterine fibroid embolization (UFE)**	Duvnjak and Andersen (2020)	Katsumori et al. (2020)
	Keyoung et al. (2001)	
	Noel-Lamy et al. (2017)	
	Zhan et al. (2005)	
**Hepatic transarterial chemoembolization (TACE)**	Abusedera et al. (2014)	Hartnell et al. (1999)
	Lee et al. (2001)	
		Molgaard et al. (1990)

Lidocaine was the anaesthetic of choice in all studies with doses ranging from 20 to 200 mg, and concentrations ranging from 0.67% to 10%. It was administered varyingly before, during or after embolisation.

The detailed characteristics of all included studies are summarised in Additional file 2: Appendix 4.

### Pain scores

#### RCTs and meta-analysis

**Fig. 2:**
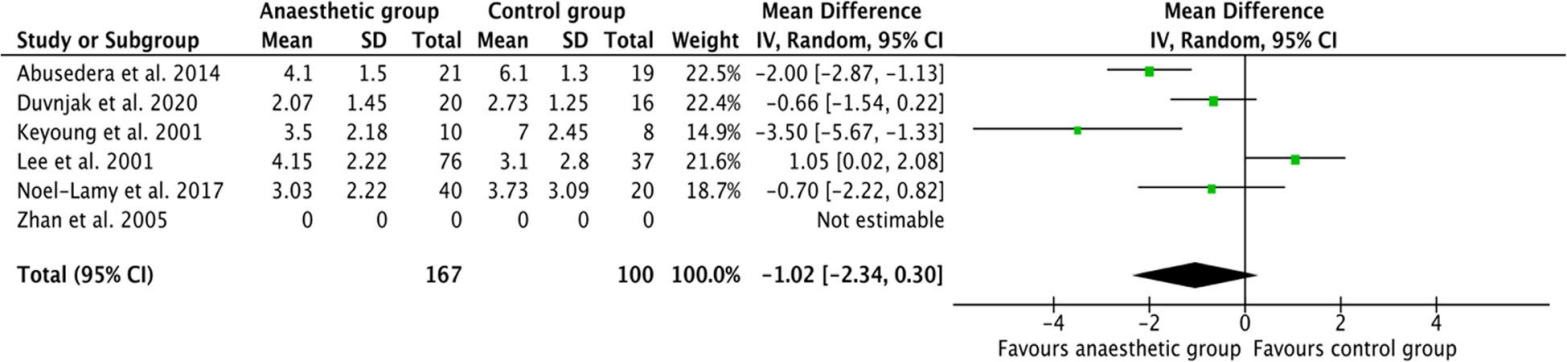
A forest plot of randomised controlled trials (RCTs) summarising the effect of intra-arterial anaesthetic on post-operative pain scores

Data from five RCTs (*n* = 267) showed that pain scores were not significantly lower in those that received anaesthetic. The pooled mean difference (PMD) in pain scores was − 1.02 (95% CI: − 2.34 to 0.30; *p* = 0.13) when comparing the study group (*n* = 167) to the control group (*n* = 100). The I^2^ statistic was 84%. The forest plot is presented in Fig. 2.

An intention-to-treat (ITT) analysis was not reported by Duvnjak and Andersen (2020), and so the per-protocol results from the study were used for the meta-analysis.

Notably, in those RCTs that reported pain scores at several time points, statistically significant reductions in pain scores were noted at 2 h by Duvnjak and Andersen (2020) (PMD: − 1.82, *p* = 0.013) and at 4 h by Noel-Lamy et al. (2017) (PMD: − 2.71, *p* < 0.001).

Zhan et al. (2005) despite being an RCT, used a qualitative 6-point pain rating scale and was therefore excluded from the meta-analysis of pain scores. A statistically significant reduction in pain (*p* < 0.01) was seen within the first 48 h post-procedure in the study group (*n* = 23) compared to the control group (*n* = 23) .

The full results are presented in Additional File 2: Appendix 5.

#### Cohort studies

One cohort study (Katsumori et al. 2020) reported pain scores over several time periods between 0 and 24 h post-procedure. However, no statistically significant differences in VAS scores were noted at any time point.

The full results are presented in Additional File 2: Appendix 6.

### Post-procedural opioid requirements

#### RCTs and meta-analysis

**Fig. 3:**
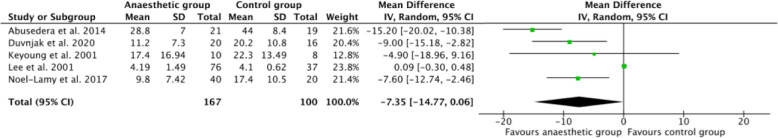
A forest plot of randomised controlled trials (RCTs) summarising the effect of intra-arterial anaesthetic on post-operative opioid requirements

Data from five RCTs (*n* = 267) showed that post-procedural opioid requirements were not significantly lower in those that received anaesthetic. The pooled mean difference was − 7.35 mg morphine equivalent (95% CI: − 14.77, 0.06; *p* = 0.05) when comparing the study group (*n* = 167) to the control group (*n* = 100). The I^2^ statistic was 93%. The forest plot is presented in Fig. 3.

An intention-to-treat (ITT) analysis was not reported by Duvnjak and Andersen (2020), and so the per-protocol results were used in the analysis.

The full results are presented in Additional File 2: Appendix 7.

#### Cohort studies

Three cohort studies reported post-procedural opioid requirements (Hartnell et al. 1999; Katsumori et al. 2020; Molgaard et al. 1990). Although two out of the three studies found a statistically significant reduction in post-operative opioid requirements, the result should be interpreted with great caution due to the limited number of studies and high risk of bias in the included studies.

Hartnell et al. (1999) recorded the total dose of opioid used in the first 24 h following the procedure. There was a statistically significant decrease in opioid use (mean difference − 26.75 mg morphine equivalent, *p* = 0.0016) when comparing the study group (*n* = 29) to the control group (*n* = 35).

Molgaard et al. (1990) reported the incidence of the requirement for a continuous morphine infusion following the embolisation procedure. There was a statistically significant decrease in the incidence of morphine infusion usage (60% lower, *p* < 0.001) in the study group (*n* = 45) than in the control group (*n* = 20).

Katsumori et al. (2020) reported the total dose of opioid administered following the embolisation procedure, although opioids were used only in the first 12 h following the procedure. There was a non-statistically significant difference in morphine dose (0.50 mg morphine equivalent [95% CI:-0.72 to 1.72], *p* = 0.42) when comparing the study group (*n* = 50) to the control group (*n* = 50).

The full results are presented in Additional file 2: Appendix 8.

### Length of hospital stay

#### RCTs

Only two RCTs recorded length of hospital stay (Abusedera et al. 2014; Noel-Lamy et al. 2017). Consequently, a meta-analysis was not deemed appropriate.

Neither study reported a statistically significant difference in the mean length of hospital stay. The mean differences in length of hospital stay were − 2.4 h (95% CI: − 27.69, 22.89; *p* = 0.85) (Abusedera et al. 2014) and − 0.9 h (95% CI: − 4.03, 2.23; *p* = 0.55) (Noel-Lamy et al. 2017) when comparing the study group to the control group.

The full results are presented in Additional file 2: Appendix 9.

#### Cohort studies

One cohort study (Hartnell et al. 1999) recorded the mean length of hospital stay of patients admitted following hepatic TACE. There was a statistically significant decrease in mean length of hospital stay (− 14 h, *p* = 0.049) when comparing the study group (*n* = 29) to the control group (*n* = 35).

The full results are presented in Additional file 2: Appendix 10.

### Complications & adverse events

Three studies reported some complications or adverse events (Keyoung et al. 2001; Lee et al. 2001; Noel-Lamy et al. 2017) associated with use of intra-arterial anaesthetic. A summary is presented in Table 2.

**Table 2:** A table summarising the complications and adverse events of all included studies

Study	Type of Study	Complications & Adverse events
Abusedera et al. 2014	RCT	None reported.
Duvnjak and Andersen 2020	RCT	None reported.
Hartnell et al. 1999	Observational (retrospective & prospective)	None reported.
Katsumori et al. 2020	Observational (retrospective)	None reported.
Keyoung et al. 2001	RCT	Trial concluded prematurely. 7/10 of those receiving lidocaine had moderate to severe vasospasm with restricted flow for 10–20 min.
Lee et al. 2001	RCT	One patient had a transient (~ 10 min) decrease in blood pressure (from 120/80 mmHg to 90/60 mmHg) after lidocaine injection.
Molgaard et al. 1990	Observational (retrospective & prospective)	None reported.
Noel-Lamy et al. 2017	RCT	Statistically significant reduction (*p* = 0.045) in proportion of patients with complete infarction of leiomyoma in those receiving lidocaine during UFE (38.9%) (*n* = 18) compared to those receiving lidocaine after UFE (77.8%) (*n* = 18) and control group (75%) (*n* = 20).
Zhan et al. 2005	RCT	None reported

Keyoung et al. (2001) intended to recruit a total of 126 patients, however, the trial was concluded prematurely as 6 of 9 patients that had received 200 mg lidocaine developed moderate to severe vasospasm that restricted flow for 10 to 20 min. As a dose-related problem had been suspected, a final patient was given 100 mg lidocaine, however they also developed significant vasospasm. This had not been noted in the control group.

Lee et al. (2001) reported a transient drop in blood pressure from 120/80 mmHg to 90/60 mmHg following lidocaine delivery, however, this was only recorded in 1 out of 76 patients that received lidocaine, and it resolved over 10 min.

Noel-Lamy et al. (2017) reported complete leiomyoma infarction at 3-month follow up in just 38.9% of patients who received lidocaine during UFE (*n* = 20), compared to 77.8% in those who received lidocaine after UFE (*n* = 20), and 75% in the control group (*n* = 20). This difference was determined to be statistically significant (*p* = 0.045) using a Kruksal-Wallis test.

### Risk of bias assessment

**Fig. 4:**
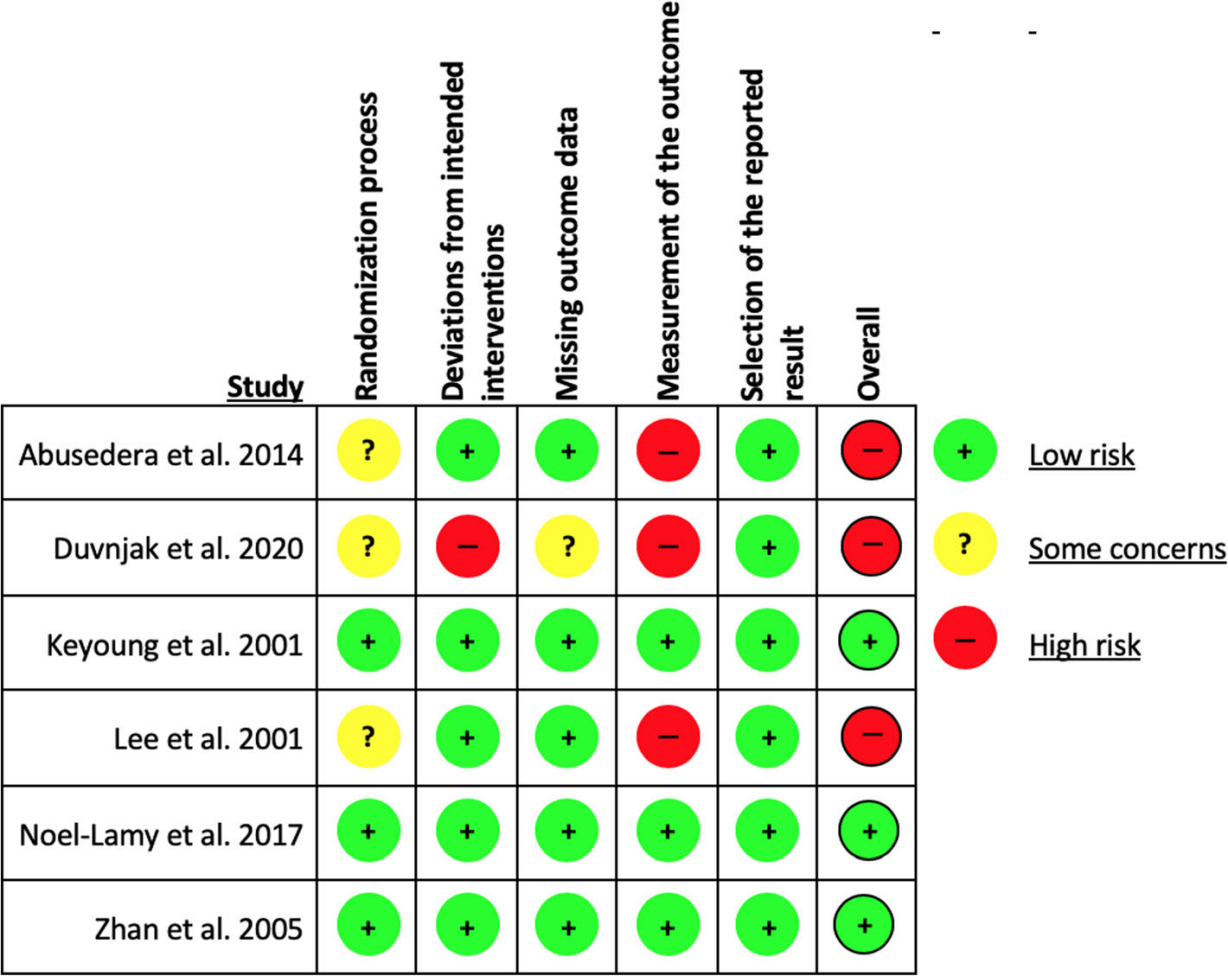
A summary of the authors’ judgements regarding the risk of bias of the RCTs included in this review, created using the RoB2 tool developed by the Cochrane Collaboration. The assessment was identical with regards to both primary outcomes

A summary of the risk of bias assessment of RCTs using the RoB 2 tool is presented in Fig. 4. The risk of bias assessment was identical for both primary outcomes. Overall, 3 studies were judged to have a high risk of bias and 3 judged to have a low risk. Of the former, all 3 were at high risk of bias with regards to the measurement of primary outcomes due to a lack of blinding, and there were some concerns regarding the randomization processes as detailed methods were not reported. There were additional concerns noted regarding missing data in Duvnjak and Andersen (2020).

A summary of the quality assessment of cohort studies using the NOS tool is presented in Table 3. Two studies were judged to be of “poor” quality (Hartnell et al. 1999; Molgaard et al. 1990) primarily due to a lack of comparability between intervention and control groups, while the remaining study was judged to be of “good” quality (Katsumori et al. 2020).

**Table 3:** A table summarising the Newcastle-Ottawa Quality Assessment Scale judgements made for the cohort studies included in this review

	Hartnell et al. 1999	Katsumori et al. 2020	Molgaard et al. 1990
**Selection**			
Representativeness of exposed cohort	*	*	
Selection of the non-exposed cohort		*	
Ascertainment of exposure	*	*	*
Demonstration that outcomes of interest were not present at start of study			*
**Comparability**			
Comparability of cohorts on the basis of the design or analysis controlled for confounders (other factors incl. Patient demographics, severity of disease, embolisation procedure)		**	
**Outcome**			
Assessment of outcome	*	*	*
Was follow-up long enough for outcomes to occur	*	*	*
Adequacy of follow-up of cohorts	*	*	*
**Total Newcastle-Ottawa Score:**	5	8	5
**AHRQ Judgement:**	Poor quality	Good quality	Poor quality

As outlined by the Newcastle-Ottawa Scale, each study was awarded a maximum of one star for each item within the Selection and Outcome categories. A maximum of two stars was given for Comparability. The total number of stars awarded to each study was translated to a qualitative judgement using conversion thresholds developed by The Agency for Healthcare Research and Quality

## Discussion

In the post-operative setting, a reduction in VAS score of 0.99 cm has been shown to be the minimum clinically significant difference (MCSD) that a patient would recognise as a decrease in pain intensity (Myles et al. 2017). The meta-analysis performed in this review suggested that mean pain scores are decreased by 1.02 (95% CI − 2.34 to 0.30; *p* = 0.13) with the use of intra-arterial anaesthesia which suggests a clinically significant difference. However, as the results are not statistically significant, the results should be interpreted with a degree of caution.

Similarly, a dose-response relationship between opioid use and adverse events is well-documented, and even a 3 mg morphine equivalent increase in opioid dose has been associated with an increase in opioid-related symptoms (Zhao et al. 2004). Therefore, the results of our meta-analyses that indicate a mean reduction in opioid dose of 7.35 mg morphine equivalent (95% CI: − 14.77, 0.06; *p* = 0.05) with intra-arterial anaesthesia suggests a clinically significant difference. However, once again, given the heterogeneity between studies and lack of statistical significance of the results, this should be interpreted with a high level of caution.

The anaesthetic effect of lidocaine (the anaesthetic used by all included studies) derives from its action as a sodium channel blocker. It delays the depolarisation of neurones and thereby inhibits pain signalling (Beecham et al. 2020). The prevailing theory for the proposed analgesic action of lidocaine in embolisation procedures was first suggested by Hartnell et al. (1999); it was theorised that lidocaine would diffuse into the local arterial wall and provide prolonged anaesthesia due to reduced blood flow and washout of the agent following embolisation. In addition, in vitro studies have shown that intra-arterial administration of lidocaine reduces the release of inflammatory mediators such as leukotriene B4 and interleukin-1 from granulocytes and mononuclear cells (Sinclair et al. 1993). Regarding TACE procedures specifically, lidocaine may also counteract the irritation caused to arteries by injection of a chemotherapeutic solution, as lidocaine has been shown to reduce pain caused by injection of contrast material during peripheral angiography (Widrich et al. 1977).

However, given that lidocaine has a half-life between 90 to 120 min (Beecham et al. 2020), it is unlikely that any analgesic effect would last up to 24 h after the procedure. This may explain why meta-analyses of mean pain scores over the first 24 h and total post-procedural opioid consumption led to results that were not statistically significant. Further study of the short-term effects of lidocaine is required.

Noel-Lamy et al. (2017) have also suggested that the injection of lidocaine after embolisation may simply lead to reflux of most of the anaesthetic, reducing its analgesic effect. Therefore, where treatment arms were combined in this analysis, the treatment effect may have been underestimated.

The studies that reported significant technical difficulties (Noel-Lamy et al. 2017; Keyoung et al. 2001) both used relatively high doses of lidocaine (200 mg) and investigated UFE specifically. The difficulties observed were associated with vasospasm induced by lidocaine; Keyoung et al. (2001) observed vasospasm directly, whilst Noel-Lamy et al. (2017) theorised that complete infarction of leiomyomata was prevented by distal vasospasm. Lidocaine is typically known to produce vasodilation and has been used to relieve vasospasm in endovascular procedures (Ishihara et al. 2015), however, there may be a dose-related effect or an effect specific to uterine arteries, as an in-vitro study showed that uterine arteries obtained from hysterectomies underwent transient dose-dependent contractions when injected with lidocaine (Cibils 1976).

Similar to this review, a previous systematic review published in 2019 assessed various pain management protocols in uterine fibroid embolisation, including intra-arterial administration of anaesthetic (Saibudeen et al. 2019). It included 3 studies on UFE and concluded that intra-uterine drug administration provided slightly better pain control compared to other analgesic protocols whilst having no significant effect on the length of hospital stay. This review provides some supportive data regarding the analgesic effect of intra-arterial anaesthetic, and also assesses the effect on post-operative opioid consumption, whilst incorporating data from additional trials and observational studies investigating both UFE and hepatic TACE.

Larger, high-quality RCTs are required to determine the utility of intra-arterial anaesthetic, particularly with regards to: the optimal timing and dosage of administration; the immediate post-operative analgesic effects; the long-term effects on hospital stay and technical success of embolization; and the importance of covariates such as the type of embolization procedure, the volume of tissue infarction, and patient comorbidities. Future studies should also aspire to report pain scores in a standardised format such as with a visual analog scale (VAS) or numerical rating scale (NRS) and should aim to provide granular data on tissue type including size of treated pathology as this would influence the degree of tissue ischaemia.

### Limitations

Individual studies often did not report or control for significant confounding factors such as patient demographics and pain or disease severity prior to embolisation. Relevant outcomes, such as length of hospital stay and post-operative nausea and vomiting were also reported inconsistently. When comparing studies, there was significant clinical heterogeneity with regards to: the method of embolisation; the dose, concentration and timing of lidocaine use; intra-operative analgesic protocols; post-operative non-opioid analgesic protocols; and the timing and method of primary outcome measurements. All of these factors may have affected the effect sizes, and this heterogeneity was likely reflected in the high I^2^ values noted in the meta-analyses. As a result, all the statistical tests that were conducted should be interpreted with caution, especially given the small number of studies (each with relatively few participants) and the high risk of bias within many of the studies, particularly the observational studies.

The general applicability of this review to all embolisation procedures is further limited by the fact that included studies only investigated UFE and hepatic TACE, with no data available for other types of embolisation procedures. The effectiveness of intra-arterial anaesthetic may be dependent upon the volume and type of tissue that is embolized, but a lack of granular data available currently precludes effective subgroup or covariate analyses.

## Conclusions

Intra-arterial anaesthetic may slightly reduce pain intensity and post-operative opioid consumption following embolisation, however the results are not statistically significant. There is very limited data available on the effect of anaesthetic on length of hospital admission, and some concerns regarding the effect of lidocaine on the technical success of embolization procedures.

There is a general lack of high-quality studies, and significant clinical heterogeneity in existing studies. High quality randomised controlled trials are required to elucidate the dose-response effect of lidocaine on opioid consumption and pain following embolisation, particularly in the first few hours post-operatively.

## Supplementary Information

**Additional file 1:** **Appendix 1.** Search strategy used for each database. Performed on 10th August. **Appendix 2.** Formula used to calculate standard deviation from confidence intervals or standard errors. **Appendix 3.** Formulae used to combine mean, standard deviation and sample sizes of studies containing 2 treatment arms.

**Additional file 2:** **Appendix 4.** A summary of the characteristics of all included studies. **Appendix 5.** A table summarising all of the randomised controlled trials that compared a quantitative pain score between intervention and control groups, with relevant results. **Appendix 6.** A table summarising the single cohort study that compared a quantitative pain score between intervention and control groups, with relevant results. **Appendix 7.** A table summarising all of the randomised controlled trials that compared post-procedural opioid requirements between intervention and control groups, with relevant results. **Appendix 8.** A table summarising the cohort studies that compared post-procedural opioid requirements between intervention and control groups, with relevant results. **Appendix 9.** A table summarising all of the randomised controlled trials that compared the length of hospital stay between intervention and control groups, with relevant results. **Appendix 10.** A table of the cohort studies that compared the length of hospital stay between intervention and control groups, with relevant results.

## Data Availability

All data generated or analysed during this study are included in this published article [and its supplementary information files].

## Abbreviations

AHRQ: Agency for Healthcare Research and Quality
CI: Confidence Interval
HCC: Hepatocellular Carcinoma
IV: Intravenous
MCSD: Minimum Clinically Significant Difference
NOS: Newcastle-Ottawa Scale
NRS: Numeric Rating Scale
PCA: Patient Controlled Analgesia
ITT: Intention-to-treat
RCT: Randomised Controlled Trial
RoB 2: Risk of Bias 2
TACE: Trans-arterial chemoembolisation
UFE: Uterine Fibroid Embolisation
VAS: Visual Analogue Scale
