# Supplementary material for: Intra-arterial anaesthetics for pain control in arterial embolisation procedures: a systematic review and meta-analysis
Source: CVIR Endovasc. 2021 Jan 5;4:6. doi: 10.1186/s42155-020-00198-z (PMC7785604; doi:10.1186/s42155-020-00198-z)
Supplement: Supplementary file 1 — Additional file 1: Appendix 1. Search strategy used for each database. Performed on 10th August. Appendix 2. Formula used to calculate standard deviation from confidence intervals or standard errors. Appendix 3. Formulae used to combine mean, standard deviation and sample sizes of studies containing 2 treatment arms. [file 42155_2020_198_MOESM1_ESM.docx]

# Additional File 1

(intraarterial OR intra-arterial OR trans-arterial OR transarterial)

AND

(anaesthesia OR anesthesia OR pain management OR pain control OR analgesia OR analgesic OR anaesthetic OR anesthetic)

AND

(embolization OR embolisation OR chemoembolization OR chemoembolization OR radioembolization OR radioembolisation)

**Appendix 1:** Search strategy used for each database. Performed on 10^th^ August 2020.

$$SD= \sqrt{N}*(upper limit-lower limit)/3.92$$

**Appendix 2:** Formula used to calculate standard deviation from confidence intervals or standard errors.


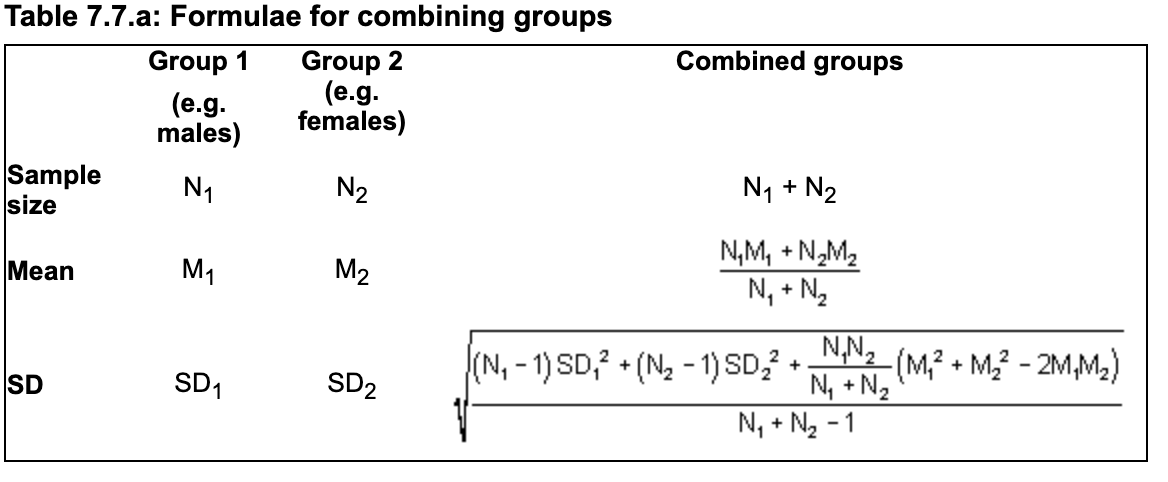


**Appendix 3:** Formulae used to combine mean, standard deviation and sample sizes of studies containing 2 treatment arms.
