# Supplementary material for: Intra-arterial anaesthetics for pain control in arterial embolisation procedures: a systematic review and meta-analysis
Source: CVIR Endovasc. 2021 Jan 5;4:6. doi: 10.1186/s42155-020-00198-z (PMC7785604; doi:10.1186/s42155-020-00198-z)
Supplement: Supplementary file 2 — Additional file 2: Appendix 4. A summary of the characteristics of all included studies. Appendix 5. A table summarising all of the randomised controlled trials that compared a quantitative pain score between intervention and control groups, with relevant results. Appendix 6. A table summarising the single cohort study that compared a quantitative pain score between intervention and control groups, with relevant results. Appendix 7. A table summarising all of the randomised controlled trials that compared post-procedural opioid requirements between intervention and control groups, with relevant results. Appendix 8. A table summarising the cohort studies that compared post-procedural opioid requirements between intervention and control groups, with relevant results. Appendix 9. A table summarising all of the randomised controlled trials that compared the length of hospital stay between intervention and control groups, with relevant results. Appendix 10. A table of the cohort studies that compared the length of hospital stay between intervention and control groups, with relevant results. [file 42155_2020_198_MOESM2_ESM.docx]

# Additional File 2

| **Study** | | **Study Design** | | | | | | | |
| --- | --- | --- | --- | --- | --- | --- | --- | --- | --- |
| **Author** | **Year** | **Type of Study** | **Embolization Procedure** | **Embolic Agent** | **Intervention** | **No. of intervention** | **Control** | **No. of control** |  |
| Abusedera et al. | 2014 | RCT | Hepatic TACE for HCC | Polyvinyl alcohol particles (PVA) (150-250 micron) | **Anaesthetic:** Buffered lidocaine, 2%  **Timing**: Before embolization  **Dose:** 100-200mg | 21 procedures, 10 patients | Saline | 19 procedures, 11 patients |  |
| Duvnjak et al. | 2020 | RCT | UFE | Tris-acryl gelatin microspheres (TAGM) (500-900 micron) | **Anaesthetic:** Lidocaine, 10%  **Timing:** After embolization  **Dose:** 200mg | 20 | No treatment | 16 (20 ITT) |  |
| Hartnell et al. | 1999 | Observational (retrospective & prospective)*^1^ | Hepatic TACE for variety of cancers | Gelfoam | **Anaesthetic:**  Lidocaine, 1%  **Timing**: before and during embolization*^3^  **Dose:** 45mg (mean), 20-80mg (range) | 29 (31 ITT) | No treatment | 35 |  |
| Katsumori et al. | 2019 | Observational (retrospective) | UFE | Tris-acryl gelatin microspheres (TAGM) (500-700 micron)*^2^ | **Anaesthetic:** Lidocaine, 1%  **Timing:** Before embolization  **Dose:** 80mg | 50 | No treatment | 50 |  |
| Keyoung et al. | 2001 | RCT | UFE | Polyvinyl alcohol particles (500-700um) | **Anaesthetic:** Lidocaine, 1%  **Timing:** Before embolization  **Dose:** 200mg (1 patient received 100mg) | 10 | Saline | 8 |  |
| Lee et al. | 2001 | RCT | Hepatic TACE for HCC | Gelfoam | **Anaesthetic:** Lidocaine, 2%  **Timing:** 1 group before embolization, 1 group after embolization  **Dose:** 100mg | Pre-TACE group: 30.  Post-TACE group: 46.  Total: 76 | No treatment | 37 |  |
| Molgaard et al. | 1990 | Observational (retrospective & prospective)*^1^ | Hepatic TACE for variety of cancers | Cross-linked collagen | **Anaesthetic:** Lidocaine, unknown %  **Timing:** 1 group before embolization, 1 group before and during embolization*^4^  **Dose:** 107mg (mean) +/- 38.4mg (SD) | Pre-TACE group: 8.  Pre- & During-TACE group: 37.  Total: 45 | No treatment | 20 |  |
| Noel-Lamy et al. | 2017 | RCT | UFE | Polyvinyl alcohol particles (PVA) (355-500 micron) | **Anaesthetic:** Lidocaine, 1%  **Timing:** 1 group during embolization, 1 group after embolization  **Dose:** 200mg | During UFE group: 20.  Post-UFE group: 20.  Total: 40 | Saline | 20 |  |
| Zhan et al. | 2005 | RCT | UFE | Polyvinyl alcohol particles (PVA) (500-710 micron) | **Anaesthetic:** Lidocaine, 0.67%  **Timing:** After embolization  **Dose:** 40mg | 23 | Saline | 23 |  |

**Appendix 4:** A summary of the characteristics of all included studies.

RCT: randomised controlled trial. UFE: uterine fibroid embolization. TACE: trans-arterial chemoembolization. HCC: hepatocellular carcinoma. ITT: intention-to-treat

*^1^ The control groups were followed retrospectively, whilst the intervention groups were followed prospectively.

*^2^ Increased to 700-900 if 6-10mL had been used and sluggish blood flow had not been obtained.

*^3^20mg lidocaine was administered before embolization, 10mg was administered up to 4 times during embolization, and 20-50mg was varyingly added to the gelfoam.

*^4^8/45 patients received 30mg boluses of lidocaine before embolization, 37/45 received an additional 60mg lidocaine added to the gelfoam.

| **Study Details** | | | | | | **Pain Score** | | | |
| --- | --- | --- | --- | --- | --- | --- | --- | --- | --- |
| **Study** | **Embolization Procedure** | **Intervention** | **No. of intervention** | **Control** | **No. of control** | **Relevant Outcome(s) Measured** | **Intervention Group (mean +/- SD) (Pain score/10 equivalent)** | **Control Group (mean +/- SD) (Pain score/10 equivalent)** | **Mean difference [95% CI]** |
| Abusedera et al. 2014 | Hepatic TACE for HCC | **Anaesthetic**: Buffered lidocaine, 2%  **Timing**:  Before embolization  **Dose**: 100-200mg | 21 procedures (10 patients) | Saline | 19 procedures (11 patients) | Mean VAS score/10 over length of admission | 4.1 +/- 1.5 | 6.1 +/- 1.3 | -2.00 [-2.87, -1.13] (p < 0.001) |
| Duvnjak et al. 2020 | UFE | **Anaesthetic**: Lidocaine, 10%  **Timing**: After embolization  **Dose**: 200mg | 20 | No treatment | 16 (20 ITT) | VAS score/100, 2h, 4h, 7h, 10h, 24h after procedure | **2h**: 4.27 +/- 2.14  **4h**: 3.97 +/- 2.31  **7h**: 4.15 +/- 2.08  **10h**: 3.47 +/- 1.96  **24h**: 2.07 +/- 1.45 | **2h**: 6.11 +/- 2.04  **4h**: 5.37 +/- 2.1  **7h**: 4.28 +/- 1.54  **10h**: 3.18 +/-1.25  **24h**: 2.73 +/- 1.25 | **2h**: -1.84 [-3.21, -0.47] (p = 0.013)  **4h**: -1.40 [-2.84, 0.04] (p = 0.069)  **7h**: -0.13 [-1.31, 1.05] (p = 0.84)  **10h**: 0.29 [-0.76, 1.34] (p = 0.61)  **24h**: -0.66 [-1.54, 0.22] (p = 0.16) |
| Keyoung et al. 2001 | UFE | **Anaesthetic:** Lidocaine, 1%  **Timing**: Before embolization  **Dose**: 200mg (1 patient received 100mg) | 10 | Saline | 8 | Maximum NRS/10 in 1st 24hrs | 3.5 +/- 2.18 | 7.0 +/- 2.45 | -3.50 [-5.67, -1.33] (p = 0.0055) |
| Lee et al. 2001 | Hepatic TACE for HCC | **Anaesthetic:** Lidocaine, 2%  **Timing:** 1 group before embolization, 1 group after embolization  **Dose:** 100mg | **Pre-TACE group:** 30.  **Post-TACE group:** 46.  **Total:** 76 | No treatment | 37 | Pain incidence.  VAS/10 morning after procedure | **Pain incidence:**  Pre-TACE group 16.7%.  Post-TACE group 38.3%.  **VAS scores:**  Pre-TACE group 3.0 +/- 2.1.  Post-TACE group: 4.9 +/- 2.0.  Combined pre/post TACE*^1^: 4.15 +/- 2.22 | **Pain incidence:** 32.4%.  **VAS score**: 3.1 +/- 2.8 | **VAS score**  Pre-TACE group: -0.10 [-1.27, 1.07] (p = 0.87)  Post-TACE group: 1.80 [0.73, 2.87] (p = 0.001)  Combined pre/post TACE**:** 1.05 [0.019, 2.08] (p = 0.033) |
| Noel-Lamy et al. 2017 | UFE | **Anaesthetic:** Lidocaine, 1%  **Timing:** 1 group during embolization, 1 group after embolization  **Dose:** 200mg | **During UFE group:** 20.  **Post-UFE group:** 20.  **Total:** 40 | Saline | 20 | VAS/100 4h, 7h, 24h after procedure | During-UFE:  **4h:** 2.86 +/- 2.45  **7h:** 3.39 +/- 2.77  **24h:** 3.66 +/- 2.81  After UFE:  **4h:** 3.58 +/- 2.26  **7h:**  3.27 +/- 2.63  **24h:** 2.39 +/- 1.21  Combined during/after UFE*^1^:  **4h**: 3.23 +/- 2.327  **7h**: 3.33 +/- 2.633  **24h**: 3.025 +/- 2.222 | **4h:** 5.94 +/- 3.03  **7h**: 4.28 +/- 2.85  **24h**: 3.73 +/- 3.09 | During-UFE:  **4h**: -3.08 [-4.79, -1.37] (p = 0.0011)  **7h**: -0.89 [-2.63, 0.85] (p = 0.32)  **24h**: -0.07 [-1.90, 1.76] (p = 0.94)  After-UFE:  **4h**: -2.36 [-4.02, -0.70] (p = 0.0082)  **7h**: -1.01 [-2.71, 0.69] (p = 0.25)  **24h**: -1.34 [-2.79, 0.11] (p = 0.079)  Combined during/after UFE:  **4h:** -2.71 [-4.22, -1.20] (p < 0.001)  **7h:** -0.95 [-2.44, 0.54] (p = 0.21)  **24h:** -0.71 [-2.22, 0.81] (p = 0.31) |
| Zhan et al. 2005 | UFE | **Anaesthetic:** Lidocaine, 0.67%  **Timing:** After embolization  **Dose:** 40mg | 23 | Saline | 23 | 6-point pain rating scale (very severe --> none) measuring pain over 5 periods: during operation, <12hrs, 12-24hrs, 24-48hrs, 48-72hrs | **During operation**: significantly reduced pain in intervention group (p<0.05)*^2^.  **<12hrs:** significantly reduced pain in intervention group (p<0.01)*^2^.  **12-24hrs:** significantly reduced pain in intervention group (p<0.01)*^2^.  **24-48hrs:** significantly reduced pain in intervention group (p<0.01)*^2^.  **48-72hrs**: no significant reduction in pain in intervention group (p>0.05)*^2^ | | |

**Appendix 5:** A table summarising all of the randomised controlled trials that compared a quantitative pain score between intervention and control groups, with relevant results.

UFE: uterine fibroid embolization. TACE: trans-arterial chemoembolization. HCC: hepatocellular carcinoma. ITT: intention to treat. VAS: visual analogue score. NRS: numeric rating scale

*^1^ The means and standard deviations from both treatment arms were combined using formulae outlined by the Cochrane Collaboration, as detailed in the methods of this review.

*^2^ These p-values were taken directly from the study as a paired Ridit test was used to determine statistical significance.

| **Study Details** | | | | | | **Pain Score** | | | |
| --- | --- | --- | --- | --- | --- | --- | --- | --- | --- |
| **Study** | **Embolization Procedure** | **Intervention** | **No. of intervention** | **Control** | **No. of control** | **Relevant Outcome(s) Measured** | **Intervention Group (mean +/- SD) (Pain Score/10 equivalent)** | **Control Group (mean +/- SD) (Pain score/10 equivalent)** | **Mean difference [95% CI]** |
| Katsumori et al. 2019 | UFE | **Anaesthetic:** Lidocaine, 1%  **Timing**: Before embolization  **Dose**: 80mg | 50 | No treatment | 50 | VAS/10 0h, 3h, 6h, 9h, 12h, 18h after procedure.  Mean VAS/10 <24hr after operation.  Max VAS/10 <24hr after operation | **0h:** 2.3 ± 2.12  **3h**: 2.2 ± 2.12  **6h**: 2.4 ± 1.41 **9h**: 1.9 ± 1.41  **12h:** 1.5 ± 1.41  **18h**: 1.0 ± 0.71 **Mean< 24h:** 1.9 ± 1.41  **Maximum < 24h**: 3.7 ± 2.12 | **0h:** 2.2 ± 2.12 **3h:** 2.9 ± 2.12  **6h:** 2.3 ± 1.41 **9h:** 1.8 ± 1.41  **12h:** 1.4 ± 1.41  **18h:** 0.9 ± 0.71 **Mean < 24h:** 1.9 ± 1.41  **Maximum < 24h:** 3.9 ± 2.12 | **0h:** 0.10 [-0.73, 0.93] (p = 0.81)  **3h:** -0.70 [-1.53, 0.13] (p = 0.10)  **6h:** 0.10 [-0.45, 0.65] (p = 0.72)  **9h:** 0.10 [-0.45, 0.65] (p = 0.72)  **12h:** 0.10 [-0.45, 0.65] (p = 0.72)  **18h:** 0.10 [-0.18, 0.38] (p = 0.48)  **Mean <24hr:** 0.00 [-0.55, 0.55] (p = 1.0)  **Maximum <24hr:** -0.20 [-1.03, 0.63] (p = 0.64) |

**Appendix 6:** A table summarising the single cohort study that compared a quantitative pain score between intervention and control groups, with relevant results. UFE: uterine fibroid embolization. VAS: visual analogue score.

| **Study Details** | | | | | | **Post-procedural opioid requirements** | | | | |
| --- | --- | --- | --- | --- | --- | --- | --- | --- | --- | --- |
| **Study** | **Embolization Procedure** | **Intervention** | **No. of intervention** | **Control** | **No. of control** | **Opioid used** | **Relevant Outcome(s) Measured** | **Intervention Group (mg morphine equivalent)** | **Control Group (mg morphine equivalent)** | **Mean difference [95% CI]** |
| Abusedera et al. 2014 | Hepatic TACE for HCC | **Anaesthetic:** Buffered lidocaine, 2%  **Timing:** Before embolization  **Dose:** 100-200mg | 21 procedures, 10 patients | Saline | 19 procedures, 11 patients | Nalbuphine | Mean daily dose of post-procedural nalbuphine.  Total dose of post-procedural nalbuphine | **Daily dose**: 8mg +/- 6.2mg  **Total dose:** 28.8mg +/- 7.0mg | **Daily dose:** 18.3mg +/- 6.2mg  **Total dose:** 44mg +/- 8.4mg | **Daily dose:** -10.30 [-14.15, -6.45] (p < 0.001)  **Total dose:** -15.20 [-20.02, -10.38] (p < 0.001) |
| Duvnjak et al. 2020 | UFE | **Anaesthetic:** Lidocaine, 10%  **Timing:** After embolization  **Dose:** 200mg | 20 | No treatment | 16 (20 ITT) | Morphine | Total dose of post-procedural morphine | **Total dose:** 11.2mg +/- 7.3mg | **Total dose:** 20.2mg +/- 10.8mg | **Total dose:** -9.00 [-15.18, -2.82] (p = 0.0053) |
| Keyoung et al. 2001 | UFE | **Anaesthetic:** Lidocaine, 1%  **Timing:** Before embolization  **Dose:** 200mg (1 patient received 100mg) | 10 | Saline | 8 | Morphine | Attempted dose of post-procedural PCA.  Given dose of post-procedural PCA.  Total dose of post-procedural PCA | **Attempted PCA dose:** 24.9mg +/- 9.11mg  **Given PCA dose:** 16.6mg +/- 13.79mg.  **Total PCA dose:** 17.4mg +/- 16.94mg | **Attempted PCA dose:** 26.3mg +/- 19.48mg  **Given PCA dose:** 17.6mg +/- 9.96mg  **Total PCA dose:** 22.3mg +/- 13.49mg | **Attempted dose:** --1.40 [-16.03, 13.23] (p = 0.84)  **Given PCA dose:** -1.00 [-11.99, 9.99] (p = 0.87)  **Total PCA dose:** -4.90 [-18.96, 9.16] (p = 0.52) |
| Lee et al. 2001 | Hepatic TACE for HCC | **Anaesthetic**: Lidocaine, 2%  **Timing:** 1  group before embolization, 1 group after embolization  **Dose:** 100mg | **Pre-TACE group**: 30  **Post-TACE group**: 46.  **Total:** 76 | No treatment | 37 | Meperidine | Total dose of post-procedural meperidine | **Total dose**:  Pre-TACE group**:** 2.5mg +/- 0.47mg  Post-TACE group**:** 5.29mg +/- 0.65mg  Combined pre/post-TACE**:** 4.19mg +/- 1.49mg* | **Total dose**: 4.1mg +/- 0.62mg | **Total dose:**  Pre-TACE group: -1.60 [-1.86, -1.34] (p<0.001)  Post-TACE group: 1.19 [0.92, 1.46] (p <0.001)  Combined pre/post TACE:  0.09 [-0.30, 0.48] (p = 0.35) |
| Noel-Lamy et al. 2017 | UFE | **Anaesthetic:** Lidocaine, 1%  **Timing:** 1 group during embolization*, 1 group after embolization  **Dose:** 200mg | **During UFE group:** 20  **Post-UFE group**: 20.  **Total:** 40 | Saline | 20 | Oxycodone and Hydromorphone | Total in-hospital narcotic dose.  Total narcotic dose in 24hrs post-procedure | **Total in-hospital narcotic dose**:  During UFE group**:** 8.5mg +/- 7.4mg.  After UFE group**:** 11.1mg +/- 9.6mg.  Combined during/after UFE: 9.8mg +/- 7.42mg*.  **Total dose in 1st 24 hours**:  During UFE group: 11.1mg +/- 9.6mg  After UFE group**:** 16.3mg +/- 11.5mg  Combined during/after UFE**:** 12.83 +/- 10.36* | **Total in-hospital narcotic dose**: 17.4mg +/- 10.5mg  **Total dose in 1st 24 hours**: 21.0mg +/- 10.5mg | **Total in-hospital narcotic dose**:  During UFE group: -8.90 [-14.53, -3.27] (p = 0.0037)  After UFE group: -6.30 [-12.54, -0.06] (p = 0.0549)  Combined during/after UFE: -7.60 [-12.74, -2.46] (p = 0.0020)  **Total dose in 1^st^ 24 hours**:  During UFE group: -9.90 [-16.14, -3.66] (p = 0.0035)  After UFE group: -4.70 [-11.52, 2.12] (p = 0.1851)  Combined during/after UFE: -8.17 [-13.78, -2.56] (p = 0.0058) |

**Appendix 7:** A table summarising all of the randomised controlled trials that compared post-procedural opioid requirements between intervention and control groups, with relevant results.

UFE: uterine fibroid embolization. TACE: trans-arterial chemoembolization. HCC: hepatocellular carcinoma. ITT: intention to treat.

*The means and standard deviations from both treatment arms were combined using formulae outlined by the Cochrane Collaboration, as detailed in the methods of this review.

| **Study Details** | | | | | | |  | | **Post-procedural opioid requirements** | | | | |
| --- | --- | --- | --- | --- | --- | --- | --- | --- | --- | --- | --- | --- | --- |
| **Study** | **Embolization Procedure** | **Intervention** | **No. of intervention** | **Control** | **No. of control** | **Opioid used** | | **Relevant Outcome(s) Measured** | | **Intervention Group (mg morphine equivalent)** | **Control Group (mg morphine equivalent)** | **Significance** |  |
| Hartnell et al. 1999 | Hepatic TACE for variety of cancers | **Anaesthetic:** Lidocaine, 1%  **Timing:** before and during embolization*^1^  **Dose:** 45mg (mean), 20-80mg (range) | 29 (31 ITT) | No treatment | 35 | Hydromorphone | | Total dose of hydromorphone in 24hrs post-procedure | | **Total dose in 1st 24hrs:**  ITT: 22.5mg (mean).  Per-protocol: 20.75mg (mean) | **Total dose in 1st 24hrs**: 47.5mg (mean) | Per-protocol: p = 0.0016 (Wilcoxon signed rank test) |  |
| Katsumori et al. 2019 | UFE | **Anaesthetic:** Lidocaine, 1%  **Timing:** Before embolization  **Dose:** 80mg | 50 | No treatment | 50 | Morphine | | Total dose of post-procedural morphine*^3^ | | **Total dose:** 13.9mg (mean) +/- 3.1mg (s.d) | **Total dose:** 13.4mg (mean) +/- 3.1mg (s.d) | p = 0.415 (paired t-test) |  |
| Molgaard et al. 1990 | Hepatic TACE for variety of cancers | **Anaesthetic:** Lidocaine, unknown %  **Timing:** 1 group before embolization, 1 group before and during embolization*^2^  **Dose:** 107mg (mean) +/- 38.4mg (SD) | **Pre-TACE group**: 8.  **Pre- & During-TACE group:** 37.  **Total:** 45 | No treatment | 20 | Morphine | | Incidence of requirement for continuous morphine infusion | | **Incidence**: 20% | **Incidence** 80% | p<0.0001 using χ^2^ analysis |  |

**Appendix 8:** A table summarising the cohort studies that compared post-procedural opioid requirements between intervention and control groups, with relevant results.

UFE: uterine fibroid embolization. TACE: trans-arterial chemoembolization. HCC: hepatocellular carcinoma. ITT: intention-to-treat. S.D: standard deviation.

*^1^20mg lidocaine was administered before embolization, 10mg was administered up to 4 times during embolization, and 20-50mg was varyingly added to the gelfoam

*^2^8/45 patients received 30mg boluses of lidocaine before embolization, 37/45 received an additional 60mg lidocaine added to the gelfoam.

*^3^Morphine was only used in the first 12 hours following the procedure, after which patients were provided with non-opioid medication.

| **Study Details** | | | | | | **Length of Hospital Stay** | | |
| --- | --- | --- | --- | --- | --- | --- | --- | --- |
| **Author** | **Embolization Procedure** | **Intervention** | **No. of intervention** | **Control** | **No. of control** | **Intervention Group** | **Control Group** | **Mean difference [95% CI]** |
| Abusedera et al. 2014 | Hepatic TACE for HCC | **Anaesthetic:**  Buffered lidocaine, 2%  **Timing**: Before embolization  **Dose:** 100-200mg | 21 procedures, 10 patients | Saline | 19 procedures, 11 patients | 88.8hrs +/- 43.2hrs | 91.2hrs +/- 38.4hrs | -2.40 [-27.69, 22.89] (p = 0.85) |
| Noel-Lamy et al. 2017 | UFE | **Anaesthetic:** Lidocaine, 1%  **Timing:** 1 group during embolization, 1 group after embolization  **Dose:** 200mg | **During UFE group**: 20.  **Post-UFE group**: 20.  **Total:** 40 | Saline | 20 | **During UAE:** 8.1hrs +/- 0.8hrs.  **After UAE**: 10.1 +/- 7.2hrs.  **Combined during/after UFE*:** 9.1 +/- 5.0263 | 10.0hrs +/- 6.2hrs | **During UAE:** -1.90 [-4.64, 0.84] (p = 0.18)  **After UAE:** 0.10 [-4.06, 4.26] (p = 0.96)  **Combined during/after UFE:** -0.90 [-4.03, 2.23] (p = 0.55) |

**Appendix 9:** A table summarising all of the randomised controlled trials that compared the length of hospital stay between intervention and control groups, with relevant results.

UFE: uterine fibroid embolization. TACE: trans-arterial chemoembolization. HCC: hepatocellular carcinoma.

*The means and standard deviations from both treatment arms were combined using formulae outlined by the Cochrane Collaboration, as detailed in the methods of this review.

| **Study Details** | | | | | | **Length of Hospital Stay** | | |
| --- | --- | --- | --- | --- | --- | --- | --- | --- |
| **Study** | **Embolization Procedure** | **Intervention** | **No. of intervention** | **Control** | **No. of control** | **Intervention Group** | **Control Group** | **Significance** |
| Hartnell et al. 1999 | Hepatic TACE for variety of cancers | **Anaesthetic:**  Lidocaine, 1%  **Timing**: before and during embolization*  **Dose:** 45mg (mean), 20-80mg (range) | 29 (31 ITT) | No treatment | 35 | 53.5hrs | 67.5hrs | p = 0.049 (Wilcoxon signed rank test) |

**Appendix 10:** A table of the cohort studies that compared the length of hospital stay between intervention and control groups, with relevant results.

UFE: uterine fibroid embolization. TACE: trans-arterial chemoembolization. HCC: hepatocellular carcinoma. ITT: intention-to-treat

*20mg lidocaine was administered before embolization, 10mg was administered up to 4 times during embolization, and 20-50mg was varyingly added to the gelfoam

H
